# Impairment of executive functions due to sleep alterations: An integrative review on the use of P300

**DOI:** 10.3389/fnins.2022.906492

**Published:** 2022-07-22

**Authors:** Nathalya Chrispim Lima, Roumen Kirov, Katie Moraes de Almondes

**Affiliations:** ^1^Postgraduate Program in Psychobiology, Federal University of Rio Grande do Norte, Natal, Brazil; ^2^Institute of Neurobiology, Bulgarian Academy of Sciences, Sofia, Bulgaria; ^3^Department of Psychology and Postgraduate Program in Psychobiology, Federal University of Rio Grande do Norte, Natal, Brazil

**Keywords:** P300, cognition, sleep, sleep deprivation, executive function, working memory, Obstructive Sleep Apnea Syndrome (OSAS), Event-Related Potentials (ERP)

## Abstract

**Objective:**

Cognitive impairment due to sleep deprivation (SD) is an important global health concern as part of the growing rates of sleep disorders and sleep deprivation worldwide. Amongst the affected cognitive processes, the effects of SD on the executive functions (EFs) show diverse methods and inconclusive or contradictory results, highlighting the importance of further research in this field. Considering this scenario, we evaluate one of the most used methods for objectively evaluating EFs on SD: the event-related potential (ERP) P300.

**Methods:**

Our study provides a comprehensive review on the use of P300 for evaluating executive functions in sleep alterations on subjects of all ages, as well as an analysis on the efficiency of P300 as an assessment method for executive functions compared to traditional neurocognitive batteries. We review the benefits of P300 application for multiple sleep/wake alterations, whether evoked in laboratory or as part of pre-existing sleep disorders.

**Results:**

We assess the diverse protocols used to elicit and complement P300, the most identified alterations in amplitude and latency, and suggest new lines of study that could benefit from P300 within the field.

**Conclusion:**

We conclude that P300 is a valuable asset for evaluating executive dysfunction under sleep deprivation both as a standalone protocol and in conjunction with subjective methods, with consistently significant results in assessing executive dysfunction in a diversity of subjects and etiologies.

## Introduction

### Sleep alterations

Sleep alterations can be described as disturbances and abnormalities on individual sleep patterns, such as difficulty in initiating sleep, sleep fragmentation, or non-restorative sleep. Those alterations may appear as part of a clinical condition (Zisapel, 2007), a sleep disorder (Tufik et al., 2010), a psychiatric disorder (Borbély and Wirz-Justice, 1982), or as lifestyle consequences (Reynolds and Banks, 2010; Shochat, 2012), and generally result in sleep deprivation (Åkerstedt, 2007).

Sleep deprivation can be divided in two subtypes: *total sleep deprivation*, characterized by episodes of vigilance equal or above 24 h and absence of 90% of REM and non-REM; and *chronic sleep restriction*, characterized by a progressive reduction of the total sleep hours over a period of time (Philip et al., 2012). Sleep deprivation has been discovered to negatively affect several cognitive processes, such as short-term memory, selective attention, and executive functioning (Krause et al., 2017), and previous literature indicates that each type is associated to different manners of cognitive impairment. Total sleep deprivation is associated with increased sleepiness, impaired memory consolidation, lapses of vigilant attention (Trošt Bobić et al., 2016), and impaired decision making (Castro and de Almondes, 2018), while chronic sleep restriction is associated with slower processing speed, selective attention lapses, and increased errors of omission (Gradisar et al., 2008; Tucker et al., 2010; Hudson et al., 2020).

Amongst the processes affected by displaced sleep, the executive functions present an intriguing range of significant, yet inconclusive findings (Gradisar et al., 2008; Tucker et al., 2010; Leite Ferreira and Moraes de Almondes, 2014; Lo et al., 2016; Honn et al., 2019; Hudson et al., 2020), with the mechanisms behind sleep-related executive dysfunction not fully understood. To better understand this difficulty and the theoretic attempts to explain it, we must first approach the different perspectives through which those functions can be explored.

### Executive functions

Friedman and Miyake (2017) describe the executive functions as “interdependent cognitive processes related to the activation of the prefrontal cortex, which are fundamental for self-regulating and goal-directed behaviors” (Friedman and Miyake, 2017). As higher-level cognitive processes, the executive functions exercise a top-down effect on behavior that allows for planning, strategizing, and working toward objectives (Miyake et al., 2000; Diamond, 2013; Friedman and Miyake, 2017), as well as inhibiting unwanted responses and ineffective strategies (Miyake et al., 2000; Friedman and Miyake, 2017). Despite their importance, there is considerable discussion in literature regarding what processes to classify as executive functions and their interactions, with different models suggested to explain their functioning (Karr et al., 2018).

Considering this on-going discussion, we draw attention to a specific model as a theoretical framework to the current review: that of Miyake et al. (2000) and Diamond (2013). The model is composed of three core processes: cognitive flexibility, working memory, and inhibitory control (which would include impulse control and selective attention) (Diamond, 2013), all with robust supporting evidence of application, measurement, and interrelation both in clinical and research trials (Friedman and Miyake, 2017). From the core functions, other complex regulating functions such as planning, creativity and decision-making would develop, thus creating a complex interrelated system of top-down control over behavior, attention, emotions, and thoughts (Diamond, 2013). Besides these core processes, other cognitive elements fundamental to proper executive functioning would be selective attention, which is directly influenced by inhibitory control and correlates with working memory, and information processing, which correlates with working memory during both development and aging (Diamond, 2013).

Two main hypotheses attempt to explain the relationship between sleep alterations and impairment of the executive functions: The Hypothesis of vigilance or lapses and waking instability (Williams et al., 1959; Doran et al., 2001) and the Frontal Lobe Hypothesis or the vulnerability of the prefrontal cortex (Horne, 1993).

The Hypothesis of Vigilance proposes that interactions between homeostatic sleep pressure and circadian propensity to sleep, both part of the Two Factor Sleep Model by Borbély and Achemann (2000) and Borbély et al. (2016), generate fluctuations in alertness while awake, destabilizing cognitive and neural performance. Such fluctuations would result in attentional lapses, temporary moments during which individuals would exhibit slower responses and deterioration of general processing during tasks that require reaction speed and surveillance (Almondes, 2019). Authors such as Durmer and Dinges (2005) and Lim and Dinges (2010) defend that attention lapses and the subsequent impairment of selective attention would relate to deficits in more complex cognitive processes, like the executive functions. However, studies relating to the three core executive functions and to short-term memory show contradictory results that this hypothesis does not fully explain.

The second hypothesis, that of Vulnerability of the Prefrontal Cortex, argues that sleep alterations affect the neurophysiological functioning of the prefrontal cortex, which relates to impairment in executive functions (Babkoff et al., 2005). The main support for this hypothesis comes from studies grounded in neuroimaging techniques, which show cortical metabolic dysfunctions associated with cognitive impairment of executive functions (Thomas et al., 2000; Killgore et al., 2006, 2008). However, other neuroimaging studies show several other areas affected by sleep alterations beyond the prefrontal cortex; thus, it is not certain if this structure is the single neurophysiological correlate to executive dysfunction (Chee and Choo, 2004; Scullin, 2017; Javaheipour et al., 2019). In addition to this, performance in more complex executive tasks, such as verbal working memory information manipulation tasks, tends to be preserved even when cortical metabolic alterations are assessed (Chee and Choo, 2004; Tucker et al., 2010). To explain these contradictory results, authors such as Staffen et al. (2002) have proposed the existence of cognitive compensatory mechanisms that could be triggered in response to increased task difficulty—which is called the Compensatory Adaptation hypothesis (Drummond and Brown, 2001; Staffen et al., 2002).

Considering the above discussion, objective studies that assess any of the three aforementioned hypothesis can have great value for our understanding of the neurophysiological and neurocognitive mechanisms behind executive dysfunction. Besides neuroimaging exams and neuropsychological tests, other possible resources in this scenario are Event-Related Potentials (ERPs), which we will detail on the following session.

### Event-related potentials: P300

Event-Related Potentials are endogenous electromagnetic wave patterns that can be measured through electroencephalography, with different patterns that can be directly correlated to vigilance, consciousness, and exposure to stimuli (Jiang et al., 2015). Another common reading and interpretation to the ERP acronym is Evoked Response Potential (Legatt, 2014), in reference to patterns that can be reliably invoked in research settings through appropriate stimuli. Of the recorded ERPs in literature, one of the most studied regarding executive functioning is the peak positive wave known as *P3* or *P300*, named as it usually occurs on a range around 300 ms after the presentation of a stimulus (Polich, 2007).

This P300 wave is assessed through latency, which relates to the time needed to process information, and through amplitude, which is related to allocation of attention by the subject (Polich, 2007). As such, P300 can be a useful resource in research concerning cognitive processes that rely heavily on attention and information processing, including the executive functions. The construct itself has been sufficiently correlated with core executive functions such as inhibitory control and working memory (Legatt, 2014; Kim et al., 2017). There is also evidence to suggest that P300 is connected to individual variations on reaction time (Ramchurn et al., 2014), which in turn connects to information processing speed; as such, this variable is also frequently considered in studies that analyze P300.

There are two subcomponents for P300 that have been identified in literature: P3a and P3b. P3b is the oldest discovered P300 subtype, associated with sustained attention and with its main source located around the parietal region. As expected in being the first discovered subtype, P3b's highest peak is at around 300 ms after the stimulus (Polich, 2007). P3a, being a later discovery, peaks a few milliseconds before P3b, at 250–280 ms. Its main source is situated on the frontal and central areas of the brain, and it is mostly associated with the recognition of recently introduced, novelty stimulus, such as a rare stimulus in an oddball paradigm task (Polich, 2007). It may also be an observed component of ERP reaction to non-target stimuli when novelty distractions are introduced, such as a dog bark amidst a number sequency (Kim et al., 2014). As the novelty stimulus becomes known, P3a amplitude is reduced (Polich, 2007). This contrasts with the so-called “NoGo P300,” where a P300 pattern is found in response exclusively to target stimuli and absent during already known distractions (Polich, 2007).

The most used method to access both P300 subtypes is through the oddball paradigm, in which the subject is instructed to discriminate a rare stimulus amidst more frequent, irrelevant stimuli. This paradigm can be administered either in auditory or visual form, and both successfully evoke P300. Between those, auditory stimulation is more commonly used and deemed more effective than visual stimulation (Polich, 2007). Alternative paradigms that can also evoke P300 include the N-back task, the Go/No-Go task, the Sternberg Working Memory Task, and the Stroop Task.

Considering the growing prevalence of sleep alterations and sleep disorders worldwide (Jahrami et al., 2021), as well as the cognitive impairment related to them, the present study intends to review pertaining literature regarding the use and efficiency of P300 to assess impairment of the executive functions under sleep deprivation on subjects of all ages. The executive functions are here understood through the model of Miyake et al. (2000) and Diamond (2013), and the term sleep alteration is here used to englobe alterations in sleep patterns brought both by sleep disorders of all etiologies and by sleep deprivation whether total or partial. We aim to summarize the previous findings on this subject throughout the years for different populations, assess the effectiveness of P300 as a tool for this objective in addition to and compared to traditional neuropsychological assessment, and propose new possibilities for studies in this developing area.

## Methodology

### Guidelines for integrative reviews, inclusion and exclusion criteria

This is an integrative review of literature, following the stage process for integrative reviews detailed by Whittemore and Knafl (2005) in their epidemiological study. The inclusion criteria for our review are the following: studies regarding sleep alterations (such as sleep deprivation, sleep restriction, and sleep disorders) that used P3a and/or P3b with the objective to assess executive functions on subjects of any age or gender. The exclusion criteria are the following: studies that did not include P3a/P3b in any form; studies that did not evaluate any of the executive functions; studies that did not evaluate sleep alterations; studies that evaluated sleep alterations as symptoms or risk factors of neurodegenerative diseases and/or other conditions that affect the executive functioning independently; and studies that were not in English.

For this study, we did not delimitate publication dates as exclusion criteria, as one of our goals is for a comprehensive review of all published data on the subject throughout the years. However, given that the present review was conducted solely through electronic databases, the access to older articles was limited, as not all of them have been digitalized.

Given that we aim to assess the effectiveness of P300 in multiple contexts and with different populations, we did not exclude articles based on the age of the participants. Although this limits the comparisons that can be made between subjects of different ages, we find this limitation necessary to conduct a review as in-depth on P300 application as possible.

### Search strategy

Five different electronic databases were selected for review: PubMed, PsychInfo, Scopus, Cochrane, and Web of Science. The keywords and descriptors selected were “sleep” OR “sleep disorder” OR “sleep alteration” OR “sleep deprivation” + “executive functions” OR “executive function” + “evoked potential” OR “p300” OR “evoked cognitive potential” OR “auditory evoked potential” OR “event related potential” OR “p3”. No further filters or descriptors were used in any of the databases.

### Study selection

The literature review for this article was conducted between May 2019 and May 2020. Studies were initially selected for further analysis by a reviewer per their abstracts and keywords, as detailed on the Search Strategy section. One hundred and twenty-seven results fitting those criteria were selected between the five databases, with the highest number of matches for all keywords being on Scopus (67 articles total). Amongst those 127 results, 19 articles were too old to be fully indexed on the databases (with only the abstracts available) or had broken links; we then attempted to contact the respective authors, or to find otherwise viable links through other indexing sites and/or the respective journal's websites. Of the 19 missing articles, 3 were excluded due to lack of viable links or contact of the authors, lowering the total number of selected articles from 127 to 124.

Of the remaining 124 studies selected, 39 were excluded for being duplicates posted in multiple databases, and 2 were excluded for not being in English, totalizing 83 articles apt for the next stage.

In the reading stage, the 83 articles were fully read through by a reviewer and searched further for the same keywords used during in the initial selection stage. In this stage, 54 studies were excluded for not addressing the research questions or properly fulfilling the inclusion criteria. The remaining 29 articles were according to the criteria and selected for the review itself.

**Figure 1:**
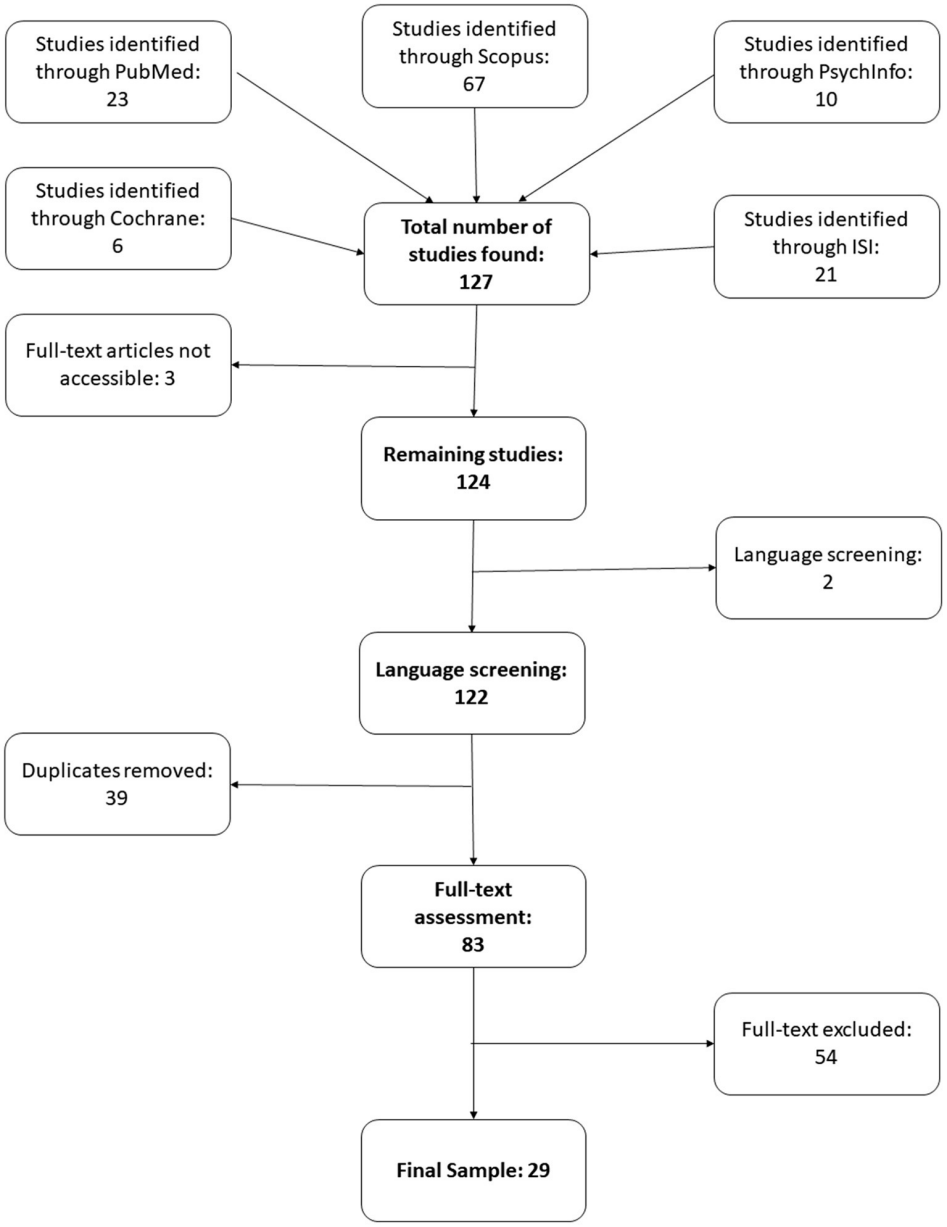
Flow chart of the search and selection stages of the review.

Each step of the review and the number of papers appraised can be seen in detail on Figure 1.

### Data collection and analysis

The 29 articles selected for the review stage had all relevant information extracted to a spreadsheet in Microsoft Excel, classified according to the following the categories: Authors and country of origin, objectives of the study, participants, study design (including EFs assessed, as we identified whether each design focused on working memory, inhibitory control, cognitive flexibility or general, non-specific executive performance), use of P300, and conclusion. Besides the information from each study, a blank space was available on the spreadsheet for further commentary on each article from the reviewer, such as clarification of any doubts or criticism of the methodology used. Any quantitative data was presented on the spreadsheet in frequency, percentage, and mean according to what was used in the original study.

## Results

### Characterization of selected studies

Twenty-nine cross-sectional studies were included in this review. Of those, 14 were case control studies, 11 were randomized control trials, and 4 were controlled clinical trials. The studies were conducted in 12 different countries, with South Korea alone being responsible for 5 articles. The United States, Turkey, and China contributed with 4 articles each. Canada produced 3 articles, Australia and India produced 2 each, and Germany, Sweden, Egypt, and Taiwan each had 1 selected article.

Considering the conditions explored, 16 studies focused on sleep disorders: 8 articles focused on Obstructive Sleep Apnea Syndrome (OSA or OSAS), including 1 article that discussed Sleep Disordered Breathing (SDB) in children; 4 articles discussed Restless Legs Syndrome (RLS), with 1 being a clinical trial for the effects of pramipexole on RSL patients; 2 articles discussed Shift Work Disorder (SWD) in regards to night shift workers; 1 article investigated inhibitory control deficits in Insomnia Disorder (ID) and another discussed the effects of modafinil on Idiopathic Hypersomnia (IH) patients.

Another 13 studies focused on both types of sleep deprivation and sleep alterations. Eight articles discussed Sleep Deprivation, with 7 employing Total Sleep Deprivation (TSD) protocols, and 1 investigating Chronic Partial Sleep Deprivation/Restriction (CPSD) in healthy subjects, while 5 articles discussed Sleep Fragmentation (SF) and subsequent SD caused by auditory stimuli.

Regarding the core EFs assessed, 11 studies discussed inhibitory control, 15 assessed working memory, and 4 explored cognitive flexibility, with 10 exploring more than one of the core EFs, or all three. Related processes, such as selective attention, were considered in 9 studies, while reaction time, a variable connected both to P300 and EFs, was considered in 7 studies. Approximately 8 studies did not disclose in detail which EFs were assessed, referring only to the general concept of executive functioning, not its underlying processes. It is important to note that there were theoretical disagreements on the concept of EFs across the selected papers: studies such as Cha et al. (2017) did not classify working memory as an executive process, but as mnesic. Others, such as Schapkin et al. (2006), described working memory as a fundamentally executive process. Such divergencies are part of the diversity of models for cognitive and executive functions in literature, and as such are to be expected and taken into consideration during this review.

### Role of P300 across selected studies

Considering the role of P300 across studies, 50% used it as an electrophysiological correlation to neurocognitive deficits, with supporting neuropsychological tests to assess those deficits. At least one study focused on comparing the efficiency of both methods as indicators of cognitive impairment Yerlikaya et al. (2018). The other 50% used only P300 components to assess impairment of the executive functions, without any supporting neuropsychological evaluation. For the type of stimulus used to elicit P300 on subjects, most studies (18 total) used visual stimulation, such as a visual Go/No Go, Visual Oddball paradigm, or N-Back task. The other 13 studies used auditory stimulation, with the great majority of those (11 total) using the classic auditory oddball paradigm. Only 2 studies opted for an auditory stop-signal task.

It is important to note the greater variability of visual methods used to elicit P300 when compared to auditory methods: a total of 8 different visual tasks vs. 2 main choices for auditory designs.

Most studies identified P3a as the third positive peak between the expected latencies of 200–300 ms, and P3b as the third positive peak between 400 and 600 ms. This is congruent with the most frequent definitions in literature (Polich, 2007). Some, such as Ko et al. (2015), considered the third peak up to 700 ms depending on the age and health of participants.

There was significant variation on average P300 latency and amplitude values between articles, which is likely related to the diversity of age and gender of participants, the presence and etiology of sleep alterations, and the methods used to evoke P300 across studies (Polich and Corey-Bloom, 2005). Most studies selected did not discriminate P300 based on sex, age, or other demographic information of the participants. Despite previous evidence in literature that there may be significant sex-related variations in P300 elicited through visual paradigms (Steffensen et al., 2008), this difference was not assessed nor provided by any of the reviewed articles. About 8 studies used single-sex samples, but even amongst those, the methodological and etiological differences between subjects were too high to properly assess differences between the samples.

Normative values of latency and amplitude of P300 during sleep alterations could not be identified due to the wide variety of etiological and methodological characteristics amongst studies, such as whether the alterations related to a sleep disorder or were caused by a sleep deprivation protocol, the number and location of electrodes, and the different stimulation procedures.

Regardless of etiology, only one study did not report any variation of P300 components related to sleep alterations: that of Cote et al. (2003). All others reported either prolonged latency (Tufik et al., 2010), reduced amplitude (Leite Ferreira and Moraes de Almondes, 2014), or both alterations (Tucker et al., 2010), either when comparing results between groups or the same participants before experimental intervention. Those same variations of P300 components were associated with impairment of the executive functions, especially inhibitory control (Renn and Cote, 2013; Zhang et al., 2014), with results on working memory variating according to etiology (Gelir et al., 2014; Jung et al., 2015). Other significant processes to the executive functioning, such as sustained attention, vigilance, and information processing also had their impairment correlated to the P300 components (Lee et al., 2003; Kusztor et al., 2019). Overall, the assorted data indicates that P300 can be a useful electrophysiological indicator of deficits to all those processes.

The most used P300 subtype was the classic P3b, with most articles (24 total) making little distinction between both, or mentioning P3a only in the introduction. Of the remaining articles, 3 defined and explored both P3a and P3b in their studies. Only 2, those of Macdonald et al. (2008) and Zhao et al. (2018) dealt exclusively with P3a. Some, such as Schapkin et al. (2007) and Qi et al. (2010) referred to P3a as P3-NoGo, for its association to the recognition of stimuli before inhibition in the No Go step of the Go/No Go task.

Further detail on all assessed studies can be seen on Table 1, including methodology and P300 application.

**Table 1:** Details of selected articles, according to following categories: authors and country of origin, objectives of the study, participants, study design, use of P300, and conclusion.

**References**	**Objectives**	**Participants**	**Sleep methods**	**Methods EFs**	**Methods P300**	**P300 Results**	**Conclusions**
**P300 and executive functions in obstructive sleep apnea**							
Ak et al. (2017) (Turkey)	• Evaluate the relationship between quality of sleep and cognitive function of Obstructive Sleep Apnea Syndrome (OSAS) patients before and after treatment.	• 30 adult and elderly OSAS patients (7 females, 33–73 age range, mean 49.63 years of age) • 30 healthy control subjects. • Age range of control subjects not disclosed in the article.	• Patients and controls filled the Epworth Sleepiness Scale and Pittsburgh Sleep Quality Scale before performing the neurocognitive tests. • Following 3 months of CPAP treatment, OSAS subjects repeated this protocol.	• Stroop test utilized to assess reaction time, attention and inhibitory control	• P300 was assessed through an auditory oddball paradigm performed after Stroop.	• OSAS patients before CPAP showed decreased P300 amplitude and increased latency when compared to control subjects (*p* = 0.041–0.048–0.058–0.000 for Fz, Cz, and Pz, respectively). • Post treatment OSAS group showed significant improvement in P300 latency (*p* = 0.000–*p* = 0.001–*p* = 0.004) and no longer significantly differed from control group. • Despite improvement in amplitude values post-CPAP, both before and after exp. group values were inferior to controls. This difference is significant for pre-CPAP and non-significant for post-CPAP (*p* = 0.314–0.544–0.450–0.198, respectively).	• OSAS patients showed: ° More complaints according to the ESS and PSQS. ° Impaired performance in the Stroop test. ° Significant P300 alterations in latency and amplitude when compared to control group.
Barnes et al. (2012) (USA)	• Identify electrophysiological signs that correlate with neurocognitive alterations in children with OSAS.	• 14 children with OSAS (9 females, age 6.21 ± 1.81 years). • 14 controls (age 6.20 ± 1.31 years, gender matched to experimental group).	• All children filled in behavioral and screening tests before undergoing polysomnography. • Questionnaires: Sleep Behavior Questionnaire (SBQ) (for sleep practices and demographic info), Childhood Symptom Inventory-4 (CSI-4) (for behavioral, emotional and cognitive symptoms) and Child Behavior Checklist (for behavioral disorders).	• Prior IQ screening performed using Peabody Picture Vocabulary Test-III (PPVT-III) as part of exclusion criteria. • Neuropsychological test battery (NEPSY) was utilized to assess attention, inhibition, and general executive functioning. • Domains utilized were Executive Function/Attention, and Memory and Learning.	• Following NEPSY, an auditory oddball task was performed to elicit ERPs. • Children had ERPs recorded during both neuropsychological assessment and oddball task.	• The experimental group showed a reverse activation pattern for P300 latency in more complex tasks, with activation in different brain regions (amygdala or temporal lobe, contrasting precuneus for controls) and a lower amplitude compared to the control group.	• Children with OSAS performed worse on neurocognitive tasks compared to the control group. • Specific differences in ERPs patterns were negatively correlated to neurocognitive performance for attention and executive functions on the NEPSY.
El-Gharib and Sharsher (2017) (Egypt)	• Investigate the influence of the CPAP machine on short term memory of patients with OSAS.	• Control Group (20 total, 10 females, mean age 47.39 ± 2.04 years, all healthy). • Study Group (20 total, 9 females, mean age 47.5 ± 9). • Study Group participants are diagnosed with OSAS through subjective and objective evaluation.	• Study Group completed a full clinical evaluation and an inventory about OSAS symptoms before and after 3 months of regular CPAP use.	• P300 is the single correlate to working memory and selective attention, with no further neurocognitive testing or protocol utilized.	• Both groups performed an auditory oddball task while recording ERPs. • P300 latency and amplitude values were compared between Study and Control. • Additional intragroup comparisons performed for Study Group before and after CPAP intervention.	• Study Group showed longer latency (296.16 ± 30.08 ms for CG and 326.62 ± 28.51 ms for SG before CPAP) and smaller P300 amplitude (7.75 ± 0.811 μV for CG and 5.32 ± 2.18 μV for SG before CPAP, p < 0.000) compared to Control Group. • Difference in amplitude between the two groups before and after CPAP use is significant • Highly significant difference between the Study Group's results before and after intervention (SG after CPAP, latency 311.57 ± 24.16 ms and amplitude 6.35 ± 1.71 μV, p < 0.000)	• P300 parameters and OSAS severity decreased after 3 months of intervention but remained below healthy population average. • This suggests benefits of the CPAP treatment not only for OSAS symptoms but also for associated cognitive impairment.
Gelir et al. (2014) (Turkey)	• Investigate the effects of OSAS on cognitive functions such as attention, learning and memory.	• 15 on OSAS Group (mean age: 41.5 ± 2.5) • 15 on Control Group (mean age: 35.9 ± 2.5).	• All participants underwent polysomnography 2 weeks prior to cognitive evaluation. • Group criteria: ° OSAS group: apnea–hypopnea index higher than 18 ° Control group: apnea-hypopnea lower than 5.	• Test battery including three visuospatial N-back tasks, a mirror-drawing task and the Trail Making Test. • N-back was utilized to assess working memory.	• P300 used as an electrophysiological correlate for selective attention and EFs. • ERPs were recorded during the first N-back task (0-back).	• P300 components lower in the OSAS group compared to the Control Group (S.E. of the difference = 1.2, t = 2.2, *p* 0.05, df = 24.8).	• Impairment in attention and executive function on OSAS subjects per P300 results. • Significantly slower reaction time than non-OSAS subjects. • No significant effects found on working memory through N-back test.
Vakulin et al. (2014) (Australia)	• Identify clinical or neurobehavioral measures capable of predicting abnormal driving associated with OSAS.	• 38 OSAS patients of differing disorder severity (10 females, age 52.0 ± 1.7 years) • 20 control subjects of matching age and gender (5 females, age 50.6 ± 2.2 years). • OSAS subjects were further classified into “resilient” or “vulnerable” depending on their performance at the simulated driving task.	• All participants underwent polysomnography to assess baseline levels 1 week prior. • General health questionnaire, the Pittsburgh Sleep Quality Index and the Epworth Sleepiness Scale. • Performed a simulated driving task on 3 weekends, under: normal sleep, restricted sleep (4 h sleep the previous night), and normal sleep + alcohol (BAC~0.05 g/dL). • During sleep restriction period, volunteers were monitored through actigraphy, sleep diary and audio messages sent to the research team prior to sleep and upon awakening.	• Stroop was utilized to measure inhibitory control and selective attention. Trail-making was utilized to assess cognitive flexibility. Symbol Digit Substitution Test (SDST) was utilized to evaluate working memory and psychomotor vigilance. • Neurocognitive scores were meant as possible predictive factors associated to abnormal driving.	• ERPs were assessed through an auditory oddball task. • P3 was characterized as the greatest positive waveform occurring 260–500 ms from stimulus onset.	• Significant difference found between OSAS and control group for in P300 latency, with OSAS subjects showing prolonged latency (328.8 ± 5.8 for Controls vs. 348.8 ± 6.2 for OSAS groups). • OSAS patients also showed reduced amplitude compared to controls (Controls: 11.8 ± 0.8 vs. OSAS Groups: 9.7 ± 0.6) though it was not statistically significant.	• OSAS patients, both vulnerable and resilient to abnormal driving, showed lower average scores than controls on all performed EFs tasks, though the difference between groups was not significant.
Whittemore and Knafl (2005) (Australia)	• Investigate the pattern of neurocognitive disfunction in OSAS.	• 50 subjects from the Brain Resource International Database (BRID), predicted to have OSAS based on the Multivariable Apnea Prediction Index (MAPI) (15 female, age: 51.59 ± 13.42 years). • 200 matched, healthy controls (60 females, age: 50.53 ± 12.83 years).	• Participants were classified as OSAS group or control group according to their scores on the MAPI questionnaire. • Depression Anxiety Stress Scale (DASS) was also applied before cognitive testing and EEG.	• Following cognitive test battery: ° Choice reaction time, ° Timing test, ° Span of visual memory, ° Digit span forward and backwards, ° Verbal interference, ° Attention switching, ° Memory recall and recognition ° Maze test.	• ERPs were recorded through standard auditory oddball paradigm. • Latency and amplitude for P300b were analyzed amongst other ERPs.	• OSAS group showed reduced P3b amplitude compared to the control group (OSAS group: 5.68 ± 0.44 vs. Controls: 7.53 ± 0.23, p < 0.0003). • No significant difference in P300 latency between groups.	• An algorithm based on demographic factors and standard OSAS symptoms was successful in selecting a group of individuals manifesting OSAS-related deficits both in information processing and in all three EFs.
Yerlikaya et al. (2018) (Turkey)	• Investigate cognitive impairment in severe OSAS patients through objective and indirect measures • Investigate the influence of hypoxemia levels on cognition.	• 54 severe OSAS patients (age: 38.94 ± 7.43, 3 female). • 34 healthy controls match in age, gender and education (age: 36.97 ± 10.83, 1 female). • OSAS patients were further divided into two smaller groups according to minimum oxygen saturation levels.	• Prior to experiment, all OSAS patients and 16 healthy controls underwent polysomnography either for classification of OSAS severity or to ensure its absence. • All volunteers filled the Epworth Sleepiness Scale (ESS). • All controls included without polysomnographic evaluation had ESS scores lower than 10.	• Mini Mental State Examination • Öktem Verbal Memory Processes Test • Wechsler Memory Scale-Revised (WMS-R) Visual Reproduction Subtest and Digit Span, • Wechsler Adult Intelligence Scale-Fourth (WAIS-IV) and Similarities Subtest, • Verbal Fluency Tests (Semantic and Lexical), • Clock Drawing Test, • Wisconsin Card Sorting Test, • Stroop Test, • Simple Copying Test • Boston Naming Test.	• A visual oddball task was used to elicit P300. • P300 is utilized as an electrophysiological correlate to executive functioning and to associated processes, including attention.	• Both low and high hypoxemia OSAS groups showed lower P300 amplitudes compared to controls [*F*_(1.86)_ = 29.315, *p* < 0.001]. • P300 amplitude difference showed a decline in parallel to increasing hypoxemia levels. The difference between both OSAS groups was not significant.	• Suggests that electrophysiological measures could be better indicators of cognitive impairment than neurocognitive tests for OSAS patients. • P300 results indicate impaired cognitive performance, while neuropsychological did not.
**P300 and executive functions in other sleep disorders**							
Belcher et al. (2015) (USA)	• Determine whether neurophysiological or occupational impairments within Shift Work Disorder were related to insomnia or excessive sleepiness as SWD symptoms.	• 34 night workers (mean age: 35.41 ± 9.35 years, 62.2% female) that had exclusively worked on night shifts for the past 6 months.	• Individuals underwent an overnight protocol composed of a multiple sleep latency test and an auditory oddball task. • On the following day, subjects completed the Endicott Work Productivity Scale (EWPS), two Insomnia Severity Indices (ISI-Day, ISI-Night) and the ESS. • Subjects were then separated between three groups: Alert Insomniacs (did not report excessive sleepiness), Sleepy Insomniacs (report excessive sleepiness) and Controls. • Objective sleepiness was assessed with a nocturnal multiple sleep latency test (MSLT).	• The EWPS includes a subscale on general executive functioning geared toward work performance, and it was utilized alongside ERPs to assess overall executive performance.	• A standard auditory oddball task was utilized for ERPs detection.	• Alert Insomniacs showed significantly lower responses on both P3a (mean difference: 1.66–1.77, *p* < 0.05) and P3b (mean difference: 1.28–1.64, *p* < 0.05) on all electrodes. • Total EWPS scores were correlated with P3a (Cpz, r = – 0.344, p < 0.05), as was the executive function subscale. • The results suggests that lower EWPS scores are more likely related to attentional lapses (associated with P3a) than to executive dysfunction per se (associated by them to P3b).	• Insomnia as a symptom was correlated with functional and cognitive impairments relating to general executive functioning, especially in relation to selective attention lapses, in subjects with Shift Work Disorder.
Cha et al. (2017) (South Korea)	• Investigate cortical mechanisms that may be involved in cognitive deficits on patients with Restless Legs Syndrome (RLS).	• 17 female patients with RLS (Age: 53.7 ± 9.6 years), not under medication • 13 healthy female volunteers (Age: 54.6 ± 7.6 years).	• Prior clinical interview and sleep questionnaire application • Questionnaires: Global Sleep Assessment Questionnaire, the Pittsburgh Sleep Quality Index (PSQI), the Epworth Sleepiness Scale (ESS), and the Insomnia Severity Index (ISI). • After ERP sessions, the Stanford Sleepiness Scale (SSS) and a visual analog scale for bothersomeness.	• P300 was assessed as a correlate of general executive function.	• P300 was assessed through a visual oddball task.	• P300 found to have a delayed latency [RLS > controls, *F*_(1,28)_ = 6.847, *p* = 0.014] and reduced amplitude [RLS < controls, *F*_(1,28)_ = 9.348, *p* = 0.005] on RLS patients compared to control group, with significant reductions in current source density and in the activation of the anterior cingulate cortex during P300 peak.	• Cognitive impairment found in RLS patients, especially of working memory, could be related to abnormal activation of the frontal region.
Choudhary et al. (2016) (India)	• Investigate attention and reaction time (visual and auditory) in night watchmen under a sleep restriction protocol.	• 50 watchmen (age: 18–35 years), divided into two groups: • Group I (n = 28) work only day time shifts and have a sleep schedule of at least 8 h per night. • Group II (*n* = 22) works exclusively nighttime shifts.	• Group II participants were sleep restricted to <3 h of sleep on a night shift in the week of the experiments. • Both groups were required to sleep full 8 h the night before the testing, and not allowed stimulants or naps during the day. • Instruments included Karolinska Sleepiness Scale (KSS) and Mood Adjective Checklist from the University of Wales Institute of Science and Technology (UWIST).	• Solely P300 was used as a correlate of selective attention performance, with an additional electric setup to record reaction times.	• P300 was assessed through an auditory oddball task.	• After the 4th and 7th day of sleep restriction, Group II participants showed prolonged P300 latency (from 300 to 400 ms to 600 ms) and P300 reduced amplitude (From slightly under 15 μV, to under 10 μV, to 5 μV) compared to Group I.	• Loss of sleep has a major impact on attention and reaction time among night shift participants, compared to daytime workers. • The authors suggest this impact on attention and processing would have a negative effect on the core EFs.
Jung et al. (2011) (South Korea)	• Identify electrophysiologic relations to cognitive dysfunction in unmedicated Restless Legs Syndrome (RLS) patients.	• 17 drug-naïve all female RLS patients (53.7 ± 9.6 years). • 13 age and gender-matched (54.6 ± 7.6) healthy controls.	• RLS patients filled in the International RLS Severity Scale (IRSL) • After EEG the session, subjects filled the Stanford Sleepiness Scale (SSS) and Bothersomeness Visual Analog Scale (VAS).	• Solely P300 was assessed as an electrophysiological correlate to attention, cognitive flexibility and information processing.	• EEG was recorded during the waking–resting state, through a visual oddball task.	• P300 latency significantly increased, and P300 amplitude significantly decreased, in the RLS group compared to controls [*F*_(1,28)_ = 14.375, *p* < 0.001 and *F*_(1,28)_ = 3.468, *p* = 0.073, respectively]. • P300 latency significantly correlated with visual bothersomeness during the ERP test (r = 0.756, p < 0.001 for frontal region; r = 0.682, *p* = 0.003 for central region; r = 0.857, p < 0.001 for parietal region).	• RLS patients show underlying cognitive impairment related to increased attention deficits and cortical/executive dysfunction when compared to control subjects.
Jung et al. (2015) (South Korea)	• Evaluate the effects of pramipexole on working memory performance of RLS patients using ERP.	• 13 drug-naive RLS patients (52.0 ± 9.48 years, 12 female).	• Prior sleep questionnaire protocol: Global Sleep Assessment Questionnaire (GSAQ), Pittsburgh Sleep Quality (PSQI), Epworth Sleepiness Scale (ESS), Beck Depression Inventory II and Insomnia Severity Index (ISI). • IRLS was used to determine RLS severity. • First ERP recording prior to start of the drug protocol. Pramipexole was then administered daily, 1 h before bed, over a period of 12 weeks. • Between 12–16 weeks after the beginning of the intervention, follow-up with another ERP recording session.	• Protocol included a visual Sternberg Item Recognition paradigm • Individual P300 amplitudes and Sternberg reaction times were compared before and after treatment.	• ERPs provoked through the Sternberg paradigm. • Both P300 and Sternberg were used as correlates to working memory.	• P300 amplitude significantly increased in the parietal area after treatment with pramipexole for all memory load (ML) sizes of the Sternberg paradigm (ML size 2, t = 3.153, *P* = 0.008, d = 0.767; ML size 3, t = 2.203, *P* = 0.048, d = 0.698; ML size 4, t = 2.483, *P* = 0.029; d = 0.693). • P300 amplitude on the frontal region significantly correlated with improvement of sleep duration in ML 2 (rho = −0.557, *P* = 0.048), and with sleep quality and sleep alterations symptoms in ML 4 (rho = −0.678, *P* = 0.011; rho = −0.592, *P* = 0.033; rho = −0.588, *P* = 0.034).	• Concludes that a daily pramipexole protocol results in improvement of working memory performance, RLS symptoms, sleep alterations and depressive symptoms in RLS patients.
Kim et al. (2014) (South Korea)	• Investigate working memory deficits in patients with Restless Legs Syndrome (RLS)	• 13 unmedicated RLS patients (11 females, age 37.5–58.0 years). • 13 healthy age-matched controls (12 females, age 45.0–54.5 years) without sleep disturbances.	• Prior polysomnography, interview and sleep questionnaire protocol to assess for inclusion and exclusion criteria. • Questionnaires: Sleep Assessment Questionnaire (GSAQ), Pittsburgh Sleep Quality Index (PSQI), Epworth Sleepiness Scale (ESS), Insomnia Severity Index (ISI), and Beck Depression Inventory II (BDI-II). • RLS group participants also filled the IRLS.	• After polysomnography, both groups completed a digit-based Sternberg working memory task.	• The Sternberg task was used to elicit P300, and both were utilized as correlates for working memory. • P300 amplitude was compared between groups.	• Significant results found in regard to brain regions (frontal, central, and parietal) and memory load (two, three, and four) as within-subject factors (F = 25.596, *P* = 0.001; F = 5.439, *P* = 0.010; F = 9.099, *P* = 0.001, respectively). • P300 amplitude was correlated with both clinical and sleep-related variables of all participants.	• Findings suggest that patients with severe RLS have working memory impairment. • Negative correlation was found between P300 amplitude and the duration of RLS on subjects, which suggests that cerebral cortical dysfunctions in patients may result from repeated RLS attacks.
Quan et al. (2013) (USA)	• Investigate the impact of Sleep Disordered Breathing (SDB) on neurocognitive functions of children 5 years after discovery of the condition.	• Experimental group: 43 children who had shown SBD at their initial exam (age: 6–11 years). • Control group: 43 children matched in age, gender and ethnicity to the experimental group.	• Participants were part of a cohort from a previous study, and had previously undergone a night of unsupervised, at-home polysomnography and filled a questionnaire regarding their sleep habits. • Re-evaluated after 5 years through the same protocol before this study.	• Sustained Working Memory Task (SWMT), designed for ERP recording. • All children also performed the Wechsler Abbreviated Scale of weeks after the SWMT. • The authors evaluated only working memory, attention and reaction times.	• P300 was used in this context as a correlate for working memory.	• Despite no significant differences in neurocognitive test performance, SDB children showed reduced P300 amplitude compared to Control group during reaction time and working memory subitems (*p* = 0.006).	• Findings suggest that SBD in children may result in long-term changes in working memory that are only detectable through objective means, and not through neurocognitive testing.
Yaman et al. (2015) (Turkey)	• Investigate the effects of modafinil on auditory P300 components on patients with idiopathic hypersomnia (IH).	• 18 patients (age range: 16–48 years) with a diagnosis of idiopathic hypersomnia (IH).	• Participants were selected amongst diagnosed IH patients from the researcher's university clinics, and thus had already gone through prior evaluation.	• P300 was utilized as the sole electrophysiological correlate to general executive functioning.	• An auditory oddball task to elicit P300 was performed by all patients before and after treatment.	• After modafinil treatment, average P300 latencies were significantly lower than latencies before the treatment on all electrode recording sites (BT: 334.9 ± 14.8 AT: 312.0 ± 26.0, p < 0.004). • An increase in amplitude was recorded at the Fz electrode site, but not at Cz or Pz.	• One week of modafinil 200 mg/day treatment improved general EFs performance, as well as general cognitive performance, and alertness of IH patients.
Zhao et al. (2018) (China)	• Investigate whether Insomnia Disorder (ID) patients show inhibitory control deficits, and to assess correlated neural mechanisms.	• 12 individuals with ID (8 females, age 49.1 ± 7.6 years). • 13 matched good sleepers (5 females, age 41.3 ± 12.4 years).	• Prior polysomnography, followed by the Pittsburgh Sleep Quality Index (PSQI), Self- • Rating Depression Scale (SDS), Self-Rating Anxiety Scale (SAS), and Barratt impulsiveness scale (BIS).	• The morning after polysomnography, all participants performed an auditory stop-signal paradigm while recording for ERPs. • Inhibitory control was assessed both through the stop-signal paradigm utilized to elicit P300 and by the ERP.	• P300 elicited through an auditory stop-sign task.	• ID patients show reduced P300 amplitude, compared to controls, during successful stop trials [*F*_(1,21)_ = 14.04, p < 0.001, η2 = 0.401].	• Findings suggest that individuals with ID show impairment to inhibitory control. • This is the first study to identify the electrophysiological correlate of those deficits as the P300 component associated with the stop stage.
**P300 and executive functions in sleep alterations**							
Cote et al. (2003) (Canada)	• Investigate brain physiology associated with sleep deprivation caused by auditory-induced sleep fragmentation.	• 8 healthy adults (4 female, mean age = 33.25 ± 6.50 years) with no sleep complaints.	• Participants spent 4 days (24 h periods) in the laboratory, with sleep fragmentation induced by auditory stimuli on nights 2 and 3. • Participants filled the Stanford Sleepiness Scale (SSS) during the test period. • Neuropsychological battery included a Profile of Mood States (POMS) (for mood) and Alpha Attenuation Task (AAT) (for sleepiness).	• Between 9:00 a.m. and 7:00 p.m., participants would complete a computerized test battery at every 2 h. • Battery: Serial addition and subtraction task (for sustained attention), Reaction time task (for reaction time), Auditory discrimination task (to elicit P300) and Perception of performance scale (for self-evaluation).	• The auditory discrimination task followed a traditional oddball paradigm to elicit P300. • P300 was used as correlate for sustained attention.	• P300 was recorded alongside N1 to assess possible effects of SD on attention. P300 results were consistent throughout the entire experiment, with no indication of impairment, which the authors attribute to possible compensatory mechanisms.	• ERP data from N1 suggests impairment of information processing, attention and alertness related to reduced arousal caused by sleep fragmentation but showed no correlation to attentional or executive impairment. P300 results indicated no impairment to EFs.
Ko et al. (2015) (Taiwan)	• Investigate the effects of sleep fragmentation on error monitoring processes.	• 13 adults (age: 20–35, gender ratio undisclosed) without any previous sleep complaints.	• Prior polysomnography and sleep questionnaire protocol composed of the Epworth Sleepiness Scale (ESS), Owl and Lark Questionnaire from Horne & Ostberg, Beck Anxiety Inventory (BAI) and Beck Depression Inventory-II (BDI-II). • 4 nights total in the lab, with a 2–3-week period washout between the first two nights and the last two. • First night would be undisturbed. Second night would induce sleep fragmentation through auditory stimulation. • Auditory stimulation could be a higher or lower setting. Participants underwent both settings after observing washout period.	• Cognitive battery composed of the CPT (sustained attention and general higher executive control), PASAT (sustained attention and information processing) and a modified flanker task (measures inhibitory control) while recording ERPs. • Performed only after SF nights.	• The modified flanker test was used to elicit P300, which was used as correlate to attention and inhibitory control alongside the flanker task.	• P300 amplitude was reduced in the high sleep fragmentation condition [SF main effect: *F*_(1,12)_ = 5.47, *p* = 0.038].	• Attention and error monitoring were impaired following one night of sleep fragmentation, regardless of total sleep hours. • Despite behavioral and neurocognitive assessment showing no significant difference between sleep deprived and non-sleep deprived phases, P300 showed a significant reduction in amplitude that could suggest an initial impairment without effects on daily functioning.
Kusztor et al. (2019) (Sweden)	• Investigate the effects of total sleep deprivation on attention and cognitive control.	• 24 healthy participants (12 females; age: 24 ± 3 years)	• Prior to testing, volunteers filled the Pittsburgh Sleep Quality Inventory (PSQI). • Participants were tested at three different periods: after a night of rest at home, after a night of rest on the lab, and after a night of total sleep deprivation on the lab, with 5 days of rest between each.	• Participants performed a visual, computerized Stop Signal task while recording ERPs. No other assessment of executive functions was used.	• P300 was assessed through the visual Stop Sign task as a correlate to selective attention and inhibitory control.	• P300 amplitude relating to the “stop” stage of the Stop Signal task was lower after the total SD night compared to the two control nights at home and at the laboratory [*F*_(2,46)_ = 14.705, η2 *p* = 0.390, *p* < 0.001], with p < 0.001 related to baseline conditions and *p* < 0.002 to maximally rested conditions.	• Higher cognitive processes, such as sustained attention, are more affected by sleep deprivation than automatic processes. • Similarly, higher functions of cognitive control are affected by sleep deprivation, while automatic functions are not.
Lee et al. (2003) (South Korea)	• Investigate cognitive impairments associated with total sleep deprivation.	• 30 healthy college students (8 females, mean age: 24.47 ± 1.33 years) without sleep complaints.	• Prior to experiment, volunteers kept a sleep diary for 2 weeks to exclude those with any prior sleep deprivation or alteration. Participants were kept awake for 38 h under continuous surveillance.	• Volunteers performed a computerized neurocognitive test battery from the Vienna Test System version IX twice a day throughout the deprivation period, at 7 a.m. and 7 p.m. • Battery included tests for vigilance, reaction time, working memory and sustained attention.	• After the neurocognitive test battery, volunteers recorded P300 through an auditory oddball test. • P300 was intended as a correlate to vigilance, reaction time and working memory.	• P300 latency was significantly longer (*F* = 32.78, *p* < 0.001), and amplitude decreased during sleep deprivation (both P3b and P3a; *F* = 17.13, *p* < 0.001; *F* = 18.28, *p* < 0.001, respectively). • Alterations on P300 components were significantly correlated with the vigilance and reaction unit tests.	• P300 alterations during total sleep deprivation can be correlated to a decrease in vigilance and a longer reaction time, but higher functions such as working memory may not be affected.
Liu et al. (2015) (China)	• Study the effects of stressful situations such as sleep deprivation and social deprivation on physiological and psychological responses of healthy subjects.	• 12 healthy male adults (age: 18–30 years)	• Participants were sorted into 72 h of social isolation or 72 h of total sleep deprivation experimental conditions. • Volunteers also filled the Profile of Mood State (POMS) and Positive Affect and Negative Affect Scale (PANAS) before and after the experimental period.	• ERPs and physiological processes were recorded while performing a numeric Go/No Go task. • Participants performed the task twice, before and after each 72 h experimental period.	• P300 was used as a correlate to inhibitory control.	• Sleep deprived participants had a lower P300 amplitude than their own pre-test conditions [*F*_(1,10)_ = 6.441, *p* = 0.029, η2 = 0.392]. • SD participants also showed lower P300 amplitude than the social isolation group [*F*_(1,10)_ = 9.66, *p* = 0.011].	• Total SD influences information processing speed, mood and vagal tone of affected individuals, resulting in impact on inhibitory control. • Sleep deprivation has more effect on P300 amplitude than social isolation.
Macdonald et al. (2008) (Canada)	• Ealuate how infrequent changes on the intensity of auditory stimuli can affect executive functioning during sleep.	• 9 healthy, self-reported good sleepers (4 females, 21.4 ± 3.0 years).	• Subjects slept at the laboratory under EEG monitoring for one night. EEG recording and initial auditory stimuli were first delivered during relaxed wakefulness while participants read a book, for control conditions. • Auditory stimuli were then interrupted to permit the subjects to naturally fall asleep, with restart 10 min after uninterrupted stage 2 sleep, and continued throughout the night.	• P300 was utilized as correlate to general executive functioning, with the specifications by the authors mainly characterizing inhibitory control.	• P300 was utilized as correlate to general executive functioning.	• Increment of auditory stimuli during the night corresponded to a peak of central P3a, with reduced amplitude when compared to the P3a registeredduring relaxed wakefulness. • Authors suggest that this reduced P3a peak corresponds to an interruption in executive functioning (defined as “central executive functioning”) during sleep, which would allow possible awareness of the external stimuli.	• Increased intensity of an acoustic stimulus during Rapid Eye Movement will elicit a P3a, presumed to correspond to the interruption of executive functioning resulting in potential awareness of stimulus input.
Molfese et al. (2013) (USA)	• Study the impact of sleep restriction on the neurocognitive skills of children, through brain imagining techniques.	• 6 male children (mean age: 7.66 years, all within the 6.6–8.3 years range), with normal sleep patterns.	• Prior polysomnography. • First week of the experiment established a 9 p.m.−7 a.m. sleep routine assessed through sleep diary and actigraphy. • Second week changed to 10 p.m.−7 a.m., depriving the children 1 h of sleep per night.	• Prior neuropsychological testing to assess their baseline levels within age average. • Neurocognitive tests performed at the lab on weekends during test period, including a speech perception task and the Stroop Naming Test for inhibitory control • ERPs recorded while testing.	• Prior baseline ERPs responses recorded and compared to results post-sleep restriction. • P300 elicited through an auditory oddball task. • P300 was utilized as a correlate of attention, inhibitory control and working memory.	• P300 amplitude significantly reduced following restricted sleep compared to baseline [*F*_(4,20)_ = 5.45, *p* < 0.0004].	• Minor sleep restriction significantly impacts children's working memory and inhibitory control, with a slower reaction on both compared to pre-experimental performance.
Qi et al. (2010) (China)	• Investigate executive functions under total sleep deprivation.	• 24 participants, all male (age: 19 ± 1.6 years) divided into two groups: Total Sleep Deprivation (TSD) group and Control group. • The abstract says 40 participants, but procedures say 24.	• Prior assessment of sleep habits two weeks before experiment through a sleep diary and Pittsburgh Sleep Quality Inventory (PSQI). • Psychological symptoms assessed through Symptom Checklist-90 and General Symptom Index. • Participants were randomly sorted into TSD or Control groups, and the TSD participants were sleep deprived under surveillance for 43 h.	• Both groups performed a visual Go/No Go task before the TSD group was submitted to SD protocol. • A second Go/No Go task was performed by both groups at 2:00 a.m. on the third day of testing while recording for ERPs.	• P300 was elicited through the Go/No Go task, and utilized as a correlate for inhibitory control, working memory and cognitive flexibility.	• The TSD group showed reduced amplitude [*F*_(2,44)_ = 7.44, p < 0.01] on sagittal sites and [*F*_(2,44)_ = 3.46, p < 0.01] on lateral sites, and a prolonged latency [*F*_(1,22)_ = 16.02, p < 0.01] than those of the control group. • Reduced amplitude is especially prominent in No Go trials than in Go trials [*F*_(1,22)_ = 3.46, *p* < 0.01].	• Significant impairment of the executive functions after total sleep deprivation, most of all working memory (for stimulus recognition) and inhibitory control (for reaction inhibition).
Ray et al. (2012) (India)	• Test the efficacy of modafinil as a countermeasure to cognitive impairment in sleep deprived individuals.	• 11 healthy males, age 25–30 years, with no history of sleep alterations or modafinil use.	• Participants were assessed through the Stanford Sleepiness Scale (SSS) and Epworth Sleepiness scale (ESS) throughout all five applications. • Sleep deprivation (SD) was monitored by researchers. • Sleep profile was assessed during experimental period through actigraphy.	• The sole method for evaluating executive functions was through ERPs. • There were five ERPs recording sessions, between 7 a.m. and 8 a.m. each day: • First session: base recordings. • Second session: after full night of SD. • Third recording: 48 h after the second, without SD. • Fourth session: 1 week after the first session, following a night of SD and intake of modafinil (400 mg/day). • Fifth session: 48 h after modafinil intake.	• ERPs components were assessed through auditory oddball paradigm.	• Significant increase in P300 latency after SD, when compared to baseline [*F*_(4,50)_ = 135.6, p < 0.01]. • Under effect of modafinil, latency significantly decreases compared to SD, with no significant changes from baseline levels [*F*_(4,50)_ = 9 5.78, p < 0.05]. • Significant increase (p < 0.001) in errors during the oddball task under SD compared to baseline, which reduced back to baseline levels after modafinil.	• Results suggest that modafinil in a dose of 400 mg/day significantly reduces attention lapses and cognitive impairment, such as working memory loss, and subjective sleepiness after SD.
Renn and Cote, 2013 (Canada)	• Evaluate the effects of total sleep deprivation on performance monitoring and inhibitory control processes.	• Total sleep deprivation group (12 females, mean age: men = 19.23 ± 1.48; women = 19.25 ± 1.29) • Control group (13 females, mean age: men-20.55 ± 2.21; women = 19.15 ± 1.57). • Total 49 participants. Groups randomly assigned post-baseline assessment.	• Prior polysomnography to assess existing SD. • All volunteers spent two consecutive nights and a day in the sleep laboratory, with first night as baseline. • On the second experimental night, SD group remained awake under surveillance of the researchers for ~34 h. • On both nights, the participants completed pre-sleep questionnaires, including the Positive and Negative Affect Scale (PANAS), Stanford Sleepiness Scale (SSS) and mood visual analog scale (VAS).	• Morning after the second experimental night, all participants were administered the Performance Assessment Batteries (PABs). • Batteries consisted of a Flanker task, Novelty Processing, Response Inhibition (Go/No Go), and 2-back working memory task.	• P300 was primarily utilized as correlate to inhibitory control and working memory. • Baseline recording performed during Flanker task and during Go/No Go.	• Sleep deprived participants showed smaller P300 amplitude compared to control group, but no significant changes in latency [*t*_(42)_ = 2.07, *p* = 0.045].	• The results support the hypothesis on the impairment of performance monitoring processes, such as inhibitory control and working memory, on sleep deprived individuals.
Schapkin et al. (2006) (Germany)	• Investigate the effects of nocturnal traffic noise on inhibitory control and cognitive performance.	• 20 healthy subjects, with no prior history of sleep complaints (11 female, age:18–30 years)	• Participants were evaluated for four nights for 3 weeks: a quiet night for baseline performance, followed three nights with aircraft noise presented at 39, 44, and 50 dBA, respectively, between 11 p.m. and 7 a.m.	• Following SF nights, volunteers performed two visual Go/No Go tasks, one considered “easy,” and another considered “difficult,” both with simultaneous ERP recording.	• P300 was primarily analyzed as correlate to inhibitory control.	• P3 latency was longer and amplitude was smaller in the difficult task, compared to the results of the easy task. • Participants classified as bad sleepers showed a smaller amplitude and longer latency for the No Go step in the difficult task, under noisy conditions [*F*_(3,54)_ = 2.88, *p* = 0.044].	• Nocturnal traffic noise impacts inhibitory control during the day, even if there is no noticeable loss of functioning.
Schapkin et al. (2006) (Germany)	• Determine the effects of noise-induced sleep disturbance on executive functions of healthy subjects, using motivational traits (“hope of success” and “fear of failure”) as mediating variables.	• 32 healthy participants total (16 female; 23 ± 3 years of age). All assessed to not have prior hearing or sight issues, nor sleep alterations. • 1 male subject's data was not used due to technical problems.	• Subjects spent four nights of three different weeks at the laboratory: one quiet control night and three nights with railway noise at different noise levels per week. • Prior to sleep and on the following morning, volunteers completed a short questionnaire on their psychological state and evaluating six parameters of sleep.	• After each night, subjects performed a task battery that included a switch task (for cognitive flexibility), a randomizing task (for working memory), an easy and difficult visual Go/No-Go task (for inhibitory control), both with simultaneous EEG recording. • Motivational traits of “hope of success” (HS) and “fear of failure” (FF) were assessed through MMG and the Achievement Orientation (AO) of the FPI.	• P300 was primarily assessed as a correlate to inhibitory control in this study design.	• P300 amplitude is significantly reduced during Noise conditions for all subjects [*F*_(3,78)_ = 3.36, *p* = 0.024]. • During No-Go trials, P300 amplitude is increased in subjects with high HS and low FF, when compared to subjects with low HS and high FF [*F*_(1,26)_ = 3.32, *p* = 0.080 and *F*_(1,24)_ = 3.41, *p* = 0.077, respectively]. • This suggests a mediating effects of motivational variables on P300 and executive control.	• Results suggest that motivation variables can modulate executive control and stimulus-response, as well as increase resistance to the negative effects of noise-induced sleep disturbance.
Zhang et al. (2014) (China)	• Investigate the possible side-effects of a zaleplon-induced nap on inhibitory control following 30 h of sleep deprivation (SD).	• 16 adult participants, all male (age: 21.8 average).	• Participants were assessed as intermediary through the Owl and Lark questionnaire prior to the experiment. • Volunteers stayed 2 consecutive days at the lab each time, with a 10-day washout interval between both experiments. • All participants were SD for 30 h and then received either zaleplon or placebo, with all of them having received both at different experiment days.	• Volunteers performed a visual Go/No Go task at five different moments throughout the experimental period: (1) At baseline; (2) after 30 h of SD; (3) after awakening from zaleplon-induced nap; (4) 4 h post-drug; and (5) 6 h post-drug.	• P300 was assessed in this design as a correlate to inhibitory control to analyze the effect of zaleplon on Go/No-Go performance.	• After 30 h of SD followed by a nap, there is significant increase in P300 latency and decrease in P300 amplitude related to the No Go step (Fz [*F*(8,120) = 49.63, *p* < 0.01], FCz [*F*_(8,120)_ = 53.71, *p* < 0.01] and Cz [*F*_(8,120)_ = 32.23, *p* < 0.01]. • No Go related P300 latencies were longer for participants who took zaleplon compared to those who took placebo. • P300 latency was shorter in the zaleplon condition compared to placebo at 4 h and 6 h post-drug (zaleplon vs. placebo, *p* < 0.01; zaleplon vs. control, *p* < 0.04; placebo vs. control, *p* < 0.01).	• These results indicate that zaleplon at a dose of 10 mg/day may help subjects maintain or recover impulse inhibition, though the side effects of zaleplon last at least 2 h post-drug.

## Discussion

### General results

At least two of the articles conclude that P300 may, in fact, be a more accurate measurement of executive functions impairment than neurocognitive testing (Kim et al., 2014; Yerlikaya et al., 2018), as it can be more sensitive to initial deficits that neurocognitive or neuropsychological batteries do not identify. However, these initial deficits did not associate with impaired functioning of the research subjects, which complicates the clinical application of P300 as a preventive method of screening for executive dysfunction outside of a research context. The absence of impaired functioning limits the potential of field professionals, such as doctors, to recognize the possibility of cognitive impairment until its onset. It is important to note as well that P300 does not discriminate impairment to a single executive process, such as solely working memory or inhibitory control, or even solely selective attention. Thus, utilizing only P300 as basis for clinical evaluation could prove limited in that regard.

Despite the above points, we consider that P300 could be a valuable tool in early diagnosis of executive dysfunction, especially if parallel to neuropsychological evaluation for a more detailed analysis of executive impairment. Considering the lower cost of EEG compared to complex neuroimaging techniques, P300 could be especially useful for the purpose of early-sign evaluation in lower income contexts.

A few of the reviewed studies seem to agree that the executive deficits evaluated would be related to lower alertness and increased processing time, rather than impairment to the mechanisms behind the executive processes themselves (Lee et al., 2003). This proposition fits with the previously mentioned *Hypothesis of vigilance or lapses and waking instability*, as a strong correlation was found between reduced P300 amplitude and delayed latency to attention deficits in several of the studies, which would point toward increased attention lapses that could affect executive functioning (Trošt Bobić et al., 2016; Castro and de Almondes, 2018). On the other hand, other studies found evidence of executive dysfunction in different etiologies being associated with abnormal functioning of frontal cortex structures (Jung et al., 2015; Cha et al., 2017), which is more in line with the *frontal lobe hypothesis* or *the vulnerability of the prefrontal cortex*, proposed by Horne (1993). These studies suggest that sleep deprivation directly affects the efficiency of the prefrontal cortex, resulting in alterations of the brain metabolism that can be visualized through EEG patterns, and thus could affect executive functioning independently of the attention pathways.

It is possible such differences could happen regarding the methodology employed by each design, as tasks that are more difficult would necessitate higher cognitive processes and automatic tasks would be more vulnerable to attention lapses due to monotony. However, this supposition would not explain all results, as discussed further below.

Both the *hypothesis of vigilance* and the *frontal lobe hypothesis* attempt to explain the executive function deficits caused by sleep alterations. However, the results pertaining to both remain mixed and inconsistent, which highlights the necessity of studies designed to further explore and test their applications.

To further discuss the results of this review, we divided the selected articles into three sessions: the first two focus on P300 and EFs in the context of sleep disorders, such as Obstructive Sleep Apnea (OSAS), Restless Legs Syndrome, Insomnia Disorder, and others, with a division between OSAS studies and studies dedicated to other disorders. A category solely for OSAS was necessary due to the sheer number of papers dedicated to this condition. The third session focuses on P300 and EFs in regard to sleep alterations, which included sleep deprivation protocols, sleep fragmentation, and sleep restriction.

This division is merely for easier understanding and discussion of P300 application for each condition by grouping similar subjects together.

### P300 and executive functions in sleep disorders

#### Obstructive sleep apnea

Obstructive Sleep Apnea Syndrome (OSAS) is a sleep disorder characterized by partial or total interruption of the upper airways during sleep, causing sleep alterations such as sleep fragmentation, morning drowsiness, and reduced vigilance. It is associated with, amongst other health issues, increased risks of cardiovascular diseases (Gonzaga et al., 2015), diabetes (Reutrakul and Mokhlesi, 2017), and psychiatric disorders (Gupta and Simpson, 2015), as well as cognitive dysfunctions. The results regarding cognitive impairment in OSAS show deficits on vigilance, attention, executive functions, psychomotor processes, and memory associated with this sleep disorder (Lal et al., 2012; Seda and Han, 2020), with considerable individual variation.

In this review, reduced amplitude was associated with executive impairment in 37.5% of studies regarding OSAS. Twenty-five found alteration in both P300 components, presenting increased latency and reduced amplitude values. One study, that of Barnes et al. (2012), explored only the increased latency.

Lower amplitude values were correlated with executive impairment even in very early stages (Wong et al., 2006; Gelir et al., 2014; Yerlikaya et al., 2018) as well as with impaired attention (Vakulin et al., 2014). El-Gharib and Sharsher (2017) found increased executive performance, as well as shorter P300 latency and increased amplitude, after 3 months of regular CPAP use by OSAS patients, though their results remained below the scores of healthy controls. Ak et al. (2017) found a similar result when comparing OSAS patients' score post-treatment with their pre-CPAP scores, and when comparing both pre- and post-CPAP scores to age and gender matched controls. Gelir et al. (2014) correlated increased latency and lower amplitude with impairments in attention and executive functions on OSAS subjects, but found no significant relations to either working or procedural memory. Interestingly, the author does not seem to consider working memory as one of the executive functions, instead classifying it only as a memory component.

Barnes et al. (2012) identified reduced latency and a lower performance in neurocognitive batteries on OSAS child patients compared to controls, as well as a reverse activation pattern on P300. Their results suggest that OSAS-affected children process information for more complex tasks in regions associated with simpler processing, such as the amygdala or temporal lobe, while healthy children process information in areas such as the precuneus, correlated with more complex processes (Zhong et al., 2013). Their results are interesting to consider in the light of the compensatory adaptation hypothesis, as it may point to the precuneus as a potential compensatory point in this stage of development.

Important to note that an executive impairment does not necessarily relate to impaired functioning. Despite most of the articles presenting significant results comparing P300 components to control groups or neuropsychological tests, not all data was significant when correlated to functions of the patient's daily life, such as driving. Vakulin et al. (2014) investigated the effects of OSAS on driving capability, as previous literature identified that OSAS patients are more at risk for traffic accidents related to morning drowsiness (George, 2007). Their assessment showed that about 60% of OSAS patients showed no significant difference from the performance of healthy controls, under either sleep deprivation or alcohol. Such results may be due to individual sensitivity to the effects of OSAS-induced sleep fragmentation, as discussed further in the P300 and Executive Functions in Sleep Deprivation session.

Regarding interesting resources for further OSAS research, Wong et al. (2006) showed the efficiency of an algorithm that could, based on demographic factors and OSAS symptoms, be a tool to select a group of individuals manifesting likely OSAS-related deficits in information processing and executive function, while Yerlikaya et al. (2018) concluded that ERPs such as P300 are more effective in detecting initial executive dysfunction in OSAS patients than neuropsychological testing.

Overall, the tendency of the surveyed articles points to P300 being an excellent resource for evaluation of OSAS patients in the research field. However, as expressed above, there are certain difficulties in implementing P300 as a clinical tool in OSAS treatment centers, as more information needs to be assessed on the reliability of P300 results for this population.

### P300 and executive functions in other sleep disorders

Considering Shift Work Disorder (SWD), both Belcher et al. (2015) and Choudhary et al. (2016) found results associating increased P300 latency and reduced amplitude to increased attention lapses and delayed reaction time in night turn workers. In line with the hypothesis of vigilance, Belcher et al. (2015) suggests that impairments to executive performance, especially in relation to shift work, likely relate to this attentional dysfunction rather than direct impairment to more complex processes such as working memory. It is important to note, however, the limitations of generalizing their results when their executive assessment relayed on the Endicott Work Productivity Scale, which is a self-application instrument not designed for in-depth cognitive evaluation (Endicott and Nee, 1997). Further research with stricter EF assessment methodology would be useful in expanding the reliability and generalization of their results.

In addition to the already discussed data, both Belcher et al. (2015) and Choudhary et al. (2016) point to the effectiveness of P300 as a tool to investigate executive dysfunction in Shift Work Disorder. However, both utilized only P300 as an assessment tool for executive dysfunction, which limits the possibilities of more detailed correlations to each of the core EFs: as P300 can be employed as correlate to either working memory or inhibitory control, for example, it is not possible to compare how each function is affected exclusively through this method. Further research comparing how P300 would correlate to accompanying neuropsychological assessment in Shift Work Disorder could be useful in this regard.

A few studies utilized P300 as an instrument while investigating cognitive deficits on patients with Restless Legs Syndrome (RLS). RLS is characterized by a compelling urge to move the legs, accompanied by an unpleasant tingling sensation, with both symptoms more common during the night (Klingelhoefer et al., 2016). Despite the motor nature of the disorder, cognitive impairments have been associated with it, especially in older patients (Klingelhoefer et al., 2016).

The P300 values on most reviewed studies show prolonged latency and reduced amplitude on RLS patients compared to control group, suggestive of cognitive impairment, as well as indication of abnormal activation of the frontal and cortical regions (Jung et al., 2011, 2015; Kim et al., 2014; Cha et al., 2017), which is relevant in the light of the vulnerability of the frontal cortex/compensatory adaptation hypothesis. In particular, Kim et al. (2014) found that impaired P300 amplitude correlated with sleep and neurocognitive results that suggest patients with severe RLS have pronounced working memory impairment that relates to such cortical dysfunctions. In another study following similar results, Jung et al. (2015) concluded that nightly doses of pramipexole before sleep significantly increased P300 amplitude post-treatment, pointing to this substance as a possible future treatment in mitigating working memory impairment for this population.

Overall, P300 is shown to be a useful resource in investigating possible frontal effects of RLS episodes in patients, as well as the effectiveness of possible treatments. Once again, more detailed studies with neuropsychological comparisons would be useful in comprehending which domains are most affected and how, especially in relation to the cortical dysfunction assessed in most of the reviewed studies. Similarly, neuroimaging studies that could trace parallels between the EEG patterns and specific area activations in this population could be useful for a better understanding of its metabolically functioning, as well as providing an easier, cheaper tool in clinical settings for diagnosis and treatment options for this group.

Regarding insomnia disorder, there was only one study eligible for this review, that of Zhao et al. (2018), which investigated whether ID patients present inhibitory control deficits and possible neural correlates. The findings suggest that individuals with ID show impairment to inhibitory control, as well as being the first study to identify an electrophysiological correlate of those deficits through P3a. A single study does not allow us to evaluate how P300 could aid in assessment and treatment of insomnia disorder. However, the results found by Ray et al. (2012) are promising and show the possibility of further applications of this methodology in future studies with this population.

Yaman et al. (2015) investigated the effects of modafinil use on P300 components of a population with Idiopathic Hypersomnia, a chronic sleep disorder that is characterized by hypersomnolence and debilitating sleep inertia (Billiard and Sonka, 2016). Their results point to a decrease in P300 latency post-treatment (Yaman et al., 2015). Similarly to ID, a single study is not enough for us to assess the effectiveness of P300 in clinical or research contexts with IH patients. However, it does suggest further possibilities for more studies with this population in the future, such as comparing baseline IH P300 latency to that of healthy controls or patients of other sleep alterations.

### P300 and executive functions in sleep deprivation

Considering the type of sleep deprivation discussed, 4 of the selected studies utilized a total sleep deprivation protocol, while 1 utilized a partial sleep deprivation protocol. Overall, 62.5% of those studies found associations between reduced P300 amplitudes and executive dysfunction under sleep deprivation, a considerably higher percentage than those that found this association for OSAS patients. This reduced amplitude correlated significantly with impairments to sustained attention (Kusztor et al., 2019), vigilance (Lee et al., 2003), and information processing (Liu et al., 2015), similarly to the results found in OSAS patients. Amongst the executive functions, inhibitory control had the most significant results (Renn and Cote, 2013; Zhang et al., 2014), with suggestions of impairment to the behavioral control mechanisms and impulse inhibition during total sleep deprivation. Results suggest that the reduced amplitude is more prominent in P3a than in P3b, at least in Go/No Go tasks (Qi et al., 2010), which would suggest more impact of total deprivation on selective attention.

Amongst selected studies, 37.5% identified longer P300 latency when compared to the participant's own performance before TSD, while one found no changes in latency compared to the control group (Renn and Cote, 2013). Kusztor et al. (2019) suggests that cognitive processes that demand more of the brain processing capacity are more affected by sleep deprivation than automatic processes, which would explain a prolonged latency. This possibility would go against the compensatory adaptation hypothesis for impairment of executive functions, as it would point to a better performance in easier tasks. Similarly, Lee et al. (2003) points that P300 alterations during total sleep deprivation can be correlated to a decrease in vigilance and a longer reaction time, which goes in line with increased attentional lapses of the hypothesis of vigilance.

On the other hand, Liu et al. (2015) found evidence that total sleep deprivation influences information processing of affected individuals, to a further extent than even extreme conditions such as complete social isolation. The authors theorize that the use of a simpler task such as Go/No-Go prevented the activation of compensatory mechanisms and allowed for a clearer understanding of the impairment, in line with the compensatory adaptation hypothesis.

Ray et al. (2012) and Zhang et al. (2014) investigated the efficacy of two substances as possible countermeasures for the effects of sleep deprivation induced cognitive impairment: modafinil and zaleplon. Drug efficiency studies such as these are useful in observing the degree of precision P300 can have in detecting executive and attentional dysfunctions in a research setting, especially concerning pharmaceutical innovation, in which such precision can be vital. They are equally useful in adding to the repertoire of successful P300 applications in provoked sleep deprivation protocols, as such protocols are intrinsically necessary in verifying drug efficiency.

Overall, the above results point to P300 being a useful tool for executive function evaluation in sleep deprivation research protocol designs, as it provides an especially sensitive objective assessment that can be reapplied and compared throughout the experimental period without the additional expenses or machinery necessary for more complex neuroimaging exams.

About partial sleep deprivation, Molfese et al. (2013) was the only article to study the impacts of sleep restriction, by sleep depriving children for 1 h before their usual bedtime for 1 week. Similar to what was found in adults, the children showed reduced P300 amplitude associated with executive dysfunction. The results suggest that minor sleep restriction already affects children's neurocognitive functioning, which is according to previous literature (Vriend et al., 2015). Again, a single paper is not enough to assess the importance of P300 as a tool in sleep restriction study designs. However, this experience highlights the successful use of P300 even in non-laboratory sleep restriction conditions, as the participants of this study returned to their own homes during the sleep restriction period instead of sleeping in a controlled environment. Despite not allowing more precise comparisons as a laboratory study would, the use of P300 in this design still permitted correlation to concurrent neuropsychological testing, thus resulting in a more in-depth, detailed evaluation of possible executive dysfunction in a wider variety of scenarios.

Another important sleep alteration related to sleep deprivation is sleep fragmentation, a sleep period intercut by brief arousals, with or without consciousness or cortical activation. While one of the core symptoms for OSAS, sleep fragmentation can be caused by a myriad of other factors and etiologies.

Schapkin and collaborators (Schapkin et al., 2006, 2007) found evidence that sleep fragmentation due to auditory interference may impact and modulate inhibitory processes, as well as that motivation variables can modulate this executive control and stimulus-response during sleep. Their results suggest that individuals with more sensitivity to sleep fragmentation show an increase in latency and reduced amplitude that would correlate to impairment of inhibitory control, especially in more difficult tasks. Such results are interesting in the light of reduced amplitude shown in studies on conditions associated with sleep fragmentation, such as OSAS (Wong et al., 2006; Gelir et al., 2014; Yerlikaya et al., 2018), and sensitivity to sleep fragmentation-related impairment could potentially explain some of the variations found amongst OSAS patients. Similarly, in Schapkin et al. (2006, 2007) no noticeable loss of functioning nor performance as assessed by neurocognitive tasks was found after sleep fragmentation episodes, with impairment shown only through ERP data, comparable to what was found by Vakulin et al. (2014) about the performance of OSAS drivers.

Macdonald et al. (2008) found comparable results to those of Schapkin et al. (2006) and Schapkin et al. (2007), in which P3a showed reduced amplitude following fragmented sleep. This result is particularly interesting, as Macdonald et al. (2008) focused on investigating the impact of SF on executive functions during wakeness after REM sleep. The authors reason that this discovery could correlate to an interruption on central executive functioning during sleep, which would allow awareness of external stimuli and possibly lead to micro-awakenings, which are once again similar, but of a significantly different etiology, to the micro-awakenings of OSAS patients.

From a different standpoint than that of Macdonald et al. (2008) and Ko et al. (2015) investigated the effects of sleep fragmentation on error monitoring processes, related to the processes of inhibitory control and working memory. Their results were independent from the total number of sleep hours, suggesting that this executive impairment is related to the fragmentation itself, and not to overall deprivation caused by arousal episodes. Their findings contrast that of Gelir et al. (2014), who found no evidence of working memory deficits in OSAS patients.

Another article, that of Cote et al. (2003), investigated the brain physiology associated with sleep deprivation caused by auditory-induced sleep fragmentation. P300 components were consistent throughout the entire experiment, with no changes in latency nor amplitude compared to baseline conditions, though processing impairment was suggested by other ERP data. The authors reason that their method for inducing sleep fragmentation could influence the stable P300 data, though the low sample size of 8 individuals and the technical difficulties during the experiment may have also played a part in the results.

## Conclusion

In conclusion, the reviewed scientific evidence suggests that there is a decrease in P300 amplitude and increase in latency under the effect of sleep alterations, both in sleep disorders and sleep deprivation. Reduced amplitude is related to executive dysfunction in OSAS patients and sleep deprived individuals; prolonged latency is also associated to impairment of executive functioning but with less conclusive evidence.

Overall, P300 has proven to be a useful tool for the evaluation of executive and attentional dysfunction in a wide variety of individuals of different ages, educational levels and socioeconomic conditions. It is shown to be an accurate measurement of executive dysfunction either alongside neurocognitive/neuropsychological tests or as the main executive assessment in a research protocol and has potential as a preventive or early sign assessment tool in clinical settings. However, unlike in traditional neuropsychological assessment, cognitive evaluation solely through P300 does not pinpoint any specific details in presentation and severity of impairment of different cognitive processes. For example, P300 testing is unable to discern executive dysfunction that affects working memory to a greater extent than inhibitory control, which could be assessed through a neuropsychological battery. This should be considered in future study designs.

This review identifies the necessity of further studies exploring the hypotheses of vigilance and frontal/compensatory adaptation in executive impairment under sleep alterations, as mixed results are associated with both throughout the assessed articles. Methodological reasons could partially explain such mixed results: across the different studies, P300 was utilized as correlate to a variety of processes such as working memory, inhibitory control, general executive functioning, and selective attention. Although there is a previous body of work to substantiate this use (Kim et al., 2014), there are no standard parameters on how to apply P300 as a correlate to each function, and no consensus amongst authors on how to evaluate and to interpret results for each of those functions. While there are attempts to adapt P300 application protocols to the focus of the study design, such as using a task like *N-back* to evoke P300 for a working memory assessment, the lack of consensus amongst authors results in difficulty to examine how P300 can be used to assess reliably and differentially such closely related processes.

Previous literature points to P300 as a correlate to information discrimination and working memory (Polich, 2007; Kim et al., 2017), with robust frontal peaks associated with P3a (Polich, 2007), and this interpretation leans toward the frontal cortex hypothesis. However, there is also strong evidence that P300 is modulated by attention, as more attentional resources on a task result in smaller P3 amplitude (Polich, 2007), which can be further related to the hypothesis of vigilance. Once more, the importance of a standard practice and consensus on the use of P300 for different cognitive processes would facilitate comparisons for both hypotheses.

There is also need for more longitudinal studies on P300's efficiency at identifying evolving executive impairment, since all studies included were cross-sectional.

We suggest that P300 may be a useful asset on studies about other related factors to sleep alterations, such as exposure to light, melatonin levels, and technology use. It could as well be useful in investigating executive dysfunctions in partial sleep deprivation, or in other conditions not contemplated here: for example, narcolepsy, in which sleep fragmentation is a common complaint (Scammell, 2015).

As limitations for this review, we can note the limited access to older studies due to the limitations of virtual databases, as well as the small number of studies included resulting in a smaller sample size and varying methodology that limits further statistical analysis. The small pool of articles also limits the results we can assess on each core EF, as not all papers discriminated between them nor detailed sufficiently their theoretical basis.

## Author contributions

NL: research, review of articles, manuscript writing, and elaborating tables and figures. KA and RK: revision and corrections. All authors contributed to the article and approved the submitted version.

## Funding

This study was supported by Coordenação de Aperfeiçoamento de Pessoal de Nível Superior - Brasil (CAPES) - Finance Code 001.

## Conflict of interest

The authors declare that the research was conducted in the absence of any commercial or financial relationships that could be construed as a potential conflict of interest.

## Publisher's note

All claims expressed in this article are solely those of the authors and do not necessarily represent those of their affiliated organizations, or those of the publisher, the editors and the reviewers. Any product that may be evaluated in this article, or claim that may be made by its manufacturer, is not guaranteed or endorsed by the publisher.
